# Intraoperative Blood Flow Evaluation Using Indocyanine Green Fluorescence Angiography for the Surgical Reconstruction of a Hilar Renal Artery Aneurysm

**DOI:** 10.3400/avd.cr.23-00085

**Published:** 2024-04-02

**Authors:** Hiromasa Nakamura, Yujiro Miura, Atsuyuki Mitsuishi, Ren Saito, Takashi Karashima, Satoshi Fukata, Hideo Fukuhara

**Affiliations:** 1 Department of Cardiovascular Surgery, Kochi University, Nankoku, Kochi, Japan; 2 Department of Urology, Kochi University, Nankoku, Kochi, Japan

**Keywords:** renal artery aneurysm, indocyanine green fluorescence angiography, surgical reconstruction

## Abstract

Surgical reconstruction is one of the standard treatments for renal artery aneurysm. However, its intraoperative evaluation is sometimes difficult depending on the operative field, aneurysm morphology, and peripheral blood vessel distribution. This case demonstrated that after renal artery reconstruction, indocyanine green fluorescence angiography is used to evaluate the results of repairing. This method is useful in visceral aneurysm evaluation not only for assessing reconstructed blood flow but also for confirming tissue perfusion of the renal parenchyma.

## Introduction

The intraoperative blood flow of reconstructed renal arteries in renal artery aneurysm (RAA) surgery can be monitored using ultrasound Doppler or a flow meter. These assessment methods are less invasive and simple. However, the evaluation becomes difficult depending on the aneurysm morphology, operative field, and peripheral blood vessel distribution. In this study, we report a case of indocyanine green (ICG) fluorescence angiography after the surgical revascularization of a hilar RAA to evaluate the reconstructed renal artery and renal parenchymal tissue perfusion.

## Case Report

**Figure 1:**
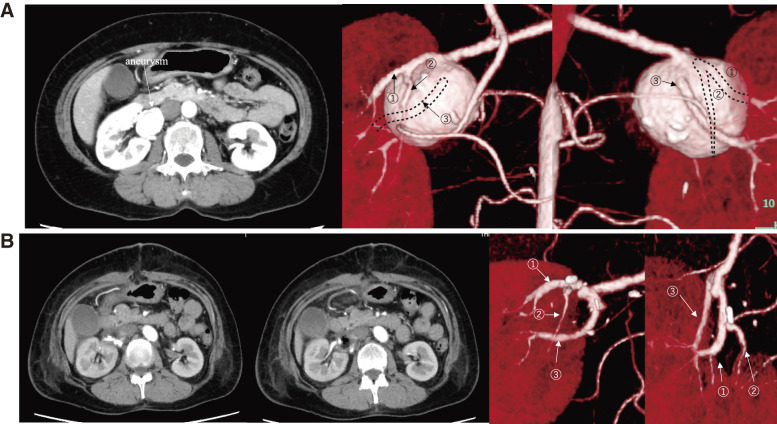
Fig. 1 Preoperative and postoperative CT. (**A**) The preoperative form of the renal aneurysm. The size of the renal aneurysm was about 32 × 35 mm (left picture). Middle and right 3D CT showed the following renal arterial flow: one inflow artery, two outflow arteries from the anterior to the aneurysm (numbers ①: superior segmental artery ②: anterior branch of the renal artery), and one outflow artery from the dorsal to the aneurysm (number ③: posterior branch of the renal artery). (**B**) postoperative CT after renal reconstruction. The axial view showed blood flow in the reconstructed artery could be confirmed. 3D CT confirmed that the outflow artery has been preserved. CT: computed tomography

A 62-year-old woman was followed up in the hospital for hypertension and liver dysfunction. She presented to the emergency department with diarrhea. The patient was 155 cm tall and weighed 60 kg. Physical examination results were uneventful. Blood tests, electrocardiogram, and chest and abdominal X-rays also showed no abnormal findings. When contrast-enhanced computed tomography (CT) was performed for close examination of diarrhea, CT showed a 32 × 35 mm saccular-type hilar RAA in the right kidney with three renal arteries outflowing from the aneurysm (**Fig. 1A**).

Endovascular treatment was initially considered, but given the morphology of the aneurysm, outflow vascular occlusion was highly possible. Considering that the aneurysm was a saccular-type RAA with a diameter of 3 cm or more and endovascular treatment was difficult, performing a surgical repair was planned.

### Operative procedure

Following a median abdominal incision, the renal portals were exposed caudally and dorsally. The right renal artery was confirmed. The inferior vena cava was also secured at cephalad and caudal, followed by exposing the renal stalk. Initially, the fatty tissue on the mass surface was dissected, and the inflow and three outflow arteries (superior segmental artery, anterior branch of renal artery, and posterior branch of the renal artery) were confirmed.

**Figure 2:**
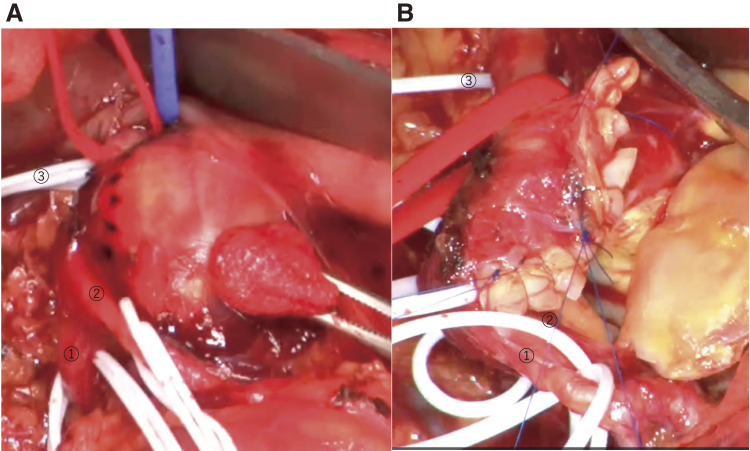
Fig. 2 Intraoperative schema, which is pre- and after-reconstruction. (**A**) The schema before reconstruction. One inflow artery (red tape), two outflow arteries from the anterior (①: superior segmental artery and ②: anterior branch of the renal artery), and one outflow artery from the dorsal were taped (③: posterior branch of the renal artery). (**B**) The schema after reconstruction. The vessel wall of the aneurysm was dissected in an island fashion. The suture line was then formed with three mattress sutures using 6-0 polypropylene, and the second layer was completed with continuous sutures. Three outflow arteries were preserved after the reconstruction.

**Figure 3:**
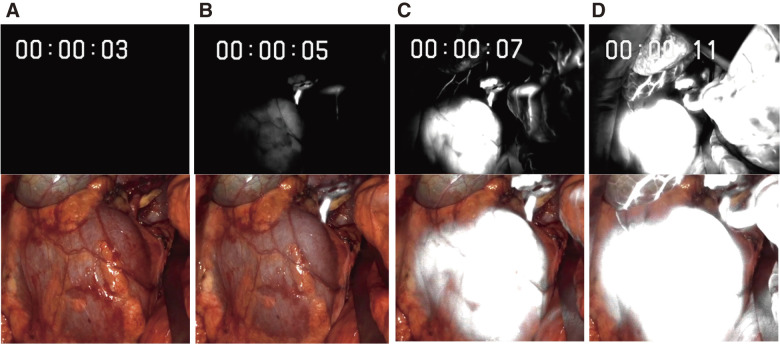
Fig. 3 The schema of the ICG fluorescence angiogram. The amount of ICG used was 2.5 mg (25 mg of ICG was dissolved in 10 mL of physiological saline, and 1 mL was used), which was administered through the central vein. Photographs were taken from a distance of 60 cm from the surgical field. (**A**) Three seconds after administration of ICG. The upper side of the kidney is slightly covered with fat. ICG has not yet reached the right kidney, and ICG fluorescence was not emitted. (**B**) 5 seconds after administration of ICG. The renal artery was being imaged, and the entire right kidney was starting to be imaged slightly on ICG. (**C**) 7 seconds after administration of ICG. The renal artery and the entire right kidney were clearly imaged with ICG. (**D**) 11 seconds after administration of ICG. The renal artery and right kidney were imaged using ICG. However, the upper border of the kidney, which was covered with fat, was not depicted. ICG: indocyanine green

After taping the inflow artery, we taped two outflow arteries located anteriorly near the calcified aneurysm and then the relatively large outflow artery present on its posterior dorsal side (**Fig. 2A**). We administered heparin and mannitol (150 mL) systemically, wrapped the kidney with an ice slush, and injected 100 mL of cold fresh blood (4°C) after clamping the inflow artery until the kidney turned white. All three outflow arteries were clamped. Next, the aneurysm was incised with the image of a vascular roll with a lumen of 3–4 mm to be reconstructed. We preserved all the inflow and outflow arteries, excised calcification entering the suture line as needed, and dissected the vessel wall in an island fashion. The suture line was then formed with three mattress sutures using 6-0 polypropylene, and the second layer was completed with continuous sutures (**Fig. 2B**). Clamping lasted for 51 minutes. Vascular bulging after reperfusion was favorable. Although the transit time flow meter (TTFM) confirmed blood flow in the two front outflow arteries, it was difficult to measure the back outflow arteries. To confirm the blood flow in the reconstructed artery and renal parenchyma, we used ICG fluorescence contrast. Entire renal parenchyma was contrasted without loss (**Fig. 3**). The postoperative course was stable, and the patient was discharged on the 12th postoperative day. Changes in creatinine (mL/min) and estimated glomerular filtration rate (eGFR: mL/min/1.73 m^2^) were not observed before and after surgery. (creatinine: 0.57 to 0.53, eGFR: 81.1 to 87.8.)

Postoperative CT (**Fig.1B**) showed an unobstructed blood flow in the inflow and outflow renal arteries.

## Discussion

In RAA, 90% of true aneurysms are outside the renal parenchyma, of which 75% are saccular-type aneurysms.^1)^ The 2020 US Vascular Surgery Guidelines state that treatment is suggested for aneurysms measuring more than 3 cm (strength of recommendation: 2 [weak]; quality of evidence: C [low]) in patients with noncomplicated RAA with an acceptable operative risk.^2)^ Endovascular treatment is indicated for most of the saccular-type aneurysms in the main trunk of visceral arteries, but surgery is required for saccular-type aneurysms with a wide aneurysmal opening that is prone to drop out even after embolization and for cases wherein the outflow artery has complex branches that are unsuitable for endovascular treatment.^3)^

In the present case, the outflow artery protruded from the aneurysm, making endovascular treatment difficult. Therefore, surgical treatment was performed. Although nephrectomy was an option, the decision was made to preserve renal function as much as possible since the patient was 62 years old and expected to have almost 30 years to live.

The revascularization methods for nonruptured RAAs are often determined by the aneurysm location. Reconstruction methods include aneurysmal suture, patch formation, aneurysmectomy, and renal artery reconstruction.^4^^,^^5)^ According to the established guidelines, RAA should be perioperatively evaluated using Doppler waveforms, which can confirm blood flow from the main trunk of the renal artery to the renal portal region and then to the renal parenchyma.^6)^

The hemodynamics of reconstructed arteries can be measured intraoperatively using ultrasound Doppler or a flow meter; both assessment tools are less invasive and simple. However, the evaluation can be challenging according to field depth, aneurysm morphology, and peripheral blood vessel distribution. Our patient’s blood flow was evaluated using a TTFM, but this method was difficult to evaluate all of the outflow arteries because of their morphology. ICG emits near-infrared fluorescence when bound to serum proteins.^7)^ ICG fluorescence angiography was reportedly used to evaluate blood flow in autologous kidney transplantation procedures.^8)^

We usually use 2.5 mg of ICG (25 mg of ICG was dissolved in 10 ml of physiological saline; of those, 1 mL was used), administered through the central vein. Photographs were taken from a distance of 60 cm from the surgical field. This method can evaluate the presence or absence and momentum of blood flow, or the size and distribution of the lumen. It can also evaluate tissue perfusion.^9)^ Therefore, ICG fluorescence angiography is a more effective method in terms of evaluating intraoperative blood flow than ultrasound Doppler; it allows visual confirmation of blood flow and inflow of blood into the renal parenchyma, which is difficult to measure using a flow meter. Conversely, ICG has some disadvantages. Firstly, ICG is difficult to quantitative evaluation. Secondly, if the area is covered with fat, it will not be visualized by ICG. In this case as well, because the upper border of the kidney was covered with fat, it was not possible to visualize that area using ICG. Thirdly, ICG is difficult to use in patients who are allergic to contrast media, but since this patient had no problem with contrast-enhanced CT, it could be used without this concern.

Yamamoto et al. also reported that ICG is useful for intraoperative blood flow evaluation in cardiovascular surgery but noted that quantitative evaluation is difficult and using ICG in combination with a TTFM is desirable.^10)^ Furthermore, there is a problem with the transmission strength of ICG: ICG can evaluate perfusion to the kidney as a whole, but it evaluates perfusion from only one direction and does not visualize the posterior portion of the kidney, so it may not be able to evaluate occlusion or stenosis of the reconstructed vessels. This is considered to be a limitation of ICG at present, and it is considered effective to combine ICG and TTFM to compensate for this limitation. In fact, in this case as well, we used TTFM to evaluate blood flow. However, due to anatomical issues, it was not possible to measure all outflow vessels using TTFM.

To solve these problems, a system for quantitative evaluation and systems capable of showing overall perfusion should be considered in the future. Nonetheless, through ICG evaluation, blood flow in the reconstructed vessels can be evaluated intraoperatively, and the distribution of blood flow to the organ parenchyma can also be easily determined. Consequently, reconstructed-vessel repair can be performed safely. In the present case, renal blood flow and blood flow distribution to the renal parenchyma were easily confirmed by ICG.

## Conclusion

In this case, we plicated a difficult-to-endovascularize hilar RAA, which was evaluated efficiently using ICG fluorescence angiography during the surgery. This imaging method is simple and allows the evaluation of blood flow and organ perfusion, making it useful in monitoring visceral aneurysms intraoperatively.

## Acknowledgments

The authors would like to thank Enago for the English language review.

## Patient Consent Statement

Appropriate informed consent was obtained from the patient to publish this case report and accompanying images.

## Disclosure Statement

All authors declare no conflicts of interest.

## Author Contributions

Study conception: MY, HN

Data collection: RS, AM, TK, SF, and HF

Analysis: none

Investigation: RS

Manuscript preparation: HN

Funding acquisition: none declared

Critical review and revision: all authors

Final approval of the article: all authors

Accountability for all aspects of the work: all authors.
